# Prognosis and Sensitivity of Adjuvant Chemotherapy in Mucinous Colorectal Adenocarcinoma without Distant Metastasis

**DOI:** 10.3390/cancers14051297

**Published:** 2022-03-02

**Authors:** Jun-Woo Bong, Jeong-An Gim, Yeonuk Ju, Chinock Cheong, Sun-Il Lee, Sang-Cheul Oh, Byung-Wook Min, Sanghee Kang

**Affiliations:** 1 Department of Surgery, Korea University Guro Hospital, Korea University College of Medicine, Seoul 08308, Korea; zest815@korea.ac.kr (J.-W.B.); snrlsnrl@korea.ac.kr (Y.J.); owho9@naver.com (C.C.); silee@korea.ac.kr (S.-I.L.); gsmin@korea.ac.kr (B.-W.M.); 2 Medical Science Research Center, College of Medicine, Korea University Guro Hospital, Seoul 08308, Korea; vitastar@korea.ac.kr; 3 Department of Oncology, Korea University Guro Hospital, Korea University College of Medicine, Seoul 08308, Korea; sachoh@korea.ac.kr

**Keywords:** colorectal cancer, mucinous adenocarcinoma, chemotherapy resistance, TCGA, consensus molecular subtype

## Abstract

**Simple Summary:**

Although chemotherapy plays an essential role in improving the survival rate of colorectal cancer, it is administered regardless of the histological classification of colorectal cancer. Mucinous adenocarcinoma is the second most common histological subtype of colorectal cancer after adenocarcinoma, accounting for 6–21% of cases. While mucinous adenocarcinoma has several poor clinical prognostic factors, controversy persists regarding its poor survival rate. The results of this study, based on analysis of the National Health Insurance database, demonstrated that mucinous adenocarcinoma has a poor survival rate related to chemoresistance. This occurs owing to the molecular properties of mucinous adenocarcinoma associated with inflammation and epithelial-mesenchymal transition (EMT). EMT, a chemoresistance-inducing pathway, increases with mucinous adenocarcinoma progression. Further studies on the development of personalized chemotherapy focusing on the molecular properties of mucinous adenocarcinoma will help improve the survival rate of patients with colorectal cancer.

**Abstract:**

In colorectal cancer, whereas mucinous adenocarcinoma (MAC) has several poor clinical prognostic factors compared to adenocarcinoma (AC), the prognosis of MAC remains controversial. We evaluated the prognosis of MAC without distant metastasis and the effects of adjuvant chemotherapy using health insurance registry data managed by South Korea. Patients with colorectal cancer between January 2014 and December 2016 were included (AC, 22,050 [96.8%]; MAC, 729 [3.2%]). We observed no difference in overall survival (OS) between AC and MAC in stages I and II. However, MAC showed a worse OS than AC in stage III disease, especially in patients administered chemotherapy (*p* < 0.001). These findings persisted after propensity score matching of clinical characteristics between AC and MAC. In addition, transcriptome analysis of The Cancer Genome Atlas (TCGA) data showed increased chemoresistance-associated pathways in MAC compared to AC. In consensus molecular subtypes (CMS) classification, unlike in AC, CMSs 1, 3, and 4 comprised most of MAC and the proportions of CMSs 3 and 4 increased with stage progression. These results suggest clues to overcome resistance to chemotherapy and develop targeted treatments in MAC.

## 1. Introduction

Colorectal cancer (CRC) is one of the most common health burdens and the second most frequent cause of cancer-related mortality worldwide [1]. The primary goal of non-metastatic CRC treatment is to cure cancer through complete resection. After surgery, adjuvant chemotherapy is recommended for patients with high-risk stage II and stage III disease, which can reduce the mortality rate by 22–32% and the recurrence rate by approximately 30% [2]. However, although adjuvant chemotherapy plays a significant role in the treatment of CRC, this decision is based solely on the pathologic TNM stage. Since the pathological and molecular subtypes of CRC have recently been reported, the relationship between these characteristics of CRC and these subtypes requires further investigation.

Mucinous adenocarcinoma (MAC) is a histologic subtype defined as adenocarcinoma (AC) with extracellular mucin pools comprising >50% of the tumor volume that occurs in 6~21% of all CRCs [3,4,5]. MAC has distinct characteristics compared to AC. MAC is more frequently located in the right colon, and the prognostic factors, including the histologic differentiation and TNM stage, are worse than those of AC [6,7]. However, the prognosis of MAC remains controversial. While most studies have reported that the prognosis of MAC is worse than that of AC [8,9,10,11], large-scale studies showed no difference between the two groups [12,13]. One reason for these contradictory results could be the very low incidence of MAC compared to that for AC, making it difficult to reach conclusions. In addition, although chemotherapy plays an important role in the prognosis of colorectal cancer, it is often difficult to provide clear information on chemotherapy based on national data [13].

This study hypothesized that if there were differences in survival rates between AC and MAC patients, the differences in the sensitivity of adjuvant chemotherapy might be the cause. Therefore, we evaluated the clinical characteristics and prognosis of AC and MAC without metastasis using the national registry and analyzed the effects of chemotherapy according to the pathologic type. In addition, we attempted to show that the sensitivity of adjuvant chemotherapy is related to molecular characteristics.

## 2. Materials and Methods

### 2.1. NQAP Data

Since 2011 in South Korea, the Health Insurance Review and Assessment Service (HIRA) has run the National Quality Assessment program (NQAP) to assess the appropriateness of treatment for major diseases and confirm appropriate healthcare expenditure [14]. We reviewed clinical data from the NQAP database. The patients in the database were diagnosed with colorectal cancer and underwent surgery between January 2014 and December 2016. The data comprised clinical characteristics including age, gender, height, weight, location of primary cancer, date, type of surgery, American Society of Anesthesiologists (ASA) classification, number of harvested lymph nodes, emergency operation, cell type, pathologic stage, resection margin status, and administration of adjuvant chemotherapy. The cell types included AC, MAC, and other types. The pathological stages were recorded according to the 7th edition of the American Joint Committee on Cancer guidelines.

### 2.2. Patients Enrolled from the NQAP Database

Patients who underwent curative resection for colorectal cancer were included and divided into AC and MAC groups. Patients with distant metastasis, other malignancies, or other cell types were excluded. Patients with insufficient data were also excluded. We analyzed differences in the clinical characteristics of both groups. Overall survival (OS) was defined as the period from the date of surgery to death. The OS in the AC and MAC groups was analyzed according to the pathologic stages. Additionally, subgroup analysis was performed according to adjuvant chemotherapy in the same pathological stages to evaluate its effects. All patients who were lost to follow-up within 30 days after surgery were excluded from the survival analysis. This study was approved by the Institutional Review Board of Korea University Guro Hospital (No. 2021GR0163), and the requirement for informed consent was waived.

### 2.3. Statistical Analysis

NQAP data were analyzed using SAS Enterprise Guide version 6.1 (SAS Institute, Cary, NC, USA) and R software version 3.5.1 (R Foundation for Statistical Computing, Vienna, Austria). Discrete values were compared using chi-squared tests, and the hazard ratios (HRs) for OS were analyzed using Cox proportional hazards regression analysis. OS was analyzed using the Kaplan-Meier method and log-rank tests. Propensity score matching was performed using the MatchIt R package to adjust for differences in baseline characteristics between the AC and MAC groups [15]. The propensity score was estimated using a multivariable logistic regression model based on age, gender, primary cancer location, body mass index (BMI), pathologic stage, emergency operation, ASA classification, resection margin status, number of harvested lymph nodes, and administration of adjuvant chemotherapy. A two-sided *p* < 0.05 was considered statistically significant.

### 2.4. Transcriptome Analysis

In addition to analyzing the clinical characteristics of MAC, the transcriptome was also analyzed to determine the molecular characteristics of MAC. The Cancer Genome Atlas (TCGA) provides information on RNA sequencing (RNASeq) data and phenotypes of CRC. The processed TCGA dataset was obtained from the Gene Expression Omnibus (GEO, accession no. GSM1536837). The phenotype and curated survival data of CRC were available in the Xena browser (https://xenabrowser.net/, accessed on 1 September 2021). Normalization (variance stabilizing transformation) and processing for pathway analysis were performed on TCGA data using the DEseq2 package [16]. A total of 610 CRC samples from TCGA had information on AC and MAC. Gene set enrichment analysis (GSEA, UC San Diego and Broad Institute, MA, USA) and Ingenuity Pathway Analysis (IPA, Qiagen, Hilden, Germany) were performed based on mRNA expression data. The consensus molecular subtype (CMS) is a representative classification that shows the molecular characteristics of CRC [17]. The CMS of 488 samples was predicted using a single-sample predictor (https://github.com/Sage-Bionetworks/CMSclassifier, accessed on 3 October 2021). Pathway analysis and CMS classification were attempted to find the differences in molecular properties between AC and MAC and reveal the tendency of the properties to change as the stage increases. For that, stages I–IV were included in the analysis. In addition, 590 TCGA CRC patients with Stage I–III CRC were analyzed for five-year survival.

## 3. Results

### 3.1. Patient Clinicopathologic Characteristics

Data from 53,217 patients who underwent surgery for colorectal cancer were collected. A total of 30,438 patients were excluded owing to incomplete data (*n* = 20,149); palliative resection (*n* = 178); other malignancies (*n* = 2316); distant metastases (*n* = 7509); and other cell types including signet ring cell carcinoma, papillary AC, tubular AC, medullary AC, and cribriform comedo-type AC (*n* = 286). Finally, the analysis included 22,779 patients with a median follow-up period of 54.5 months. The AC and MAC groups included 22,050 (96.8%) and 729 (3.2%) patients, respectively. The characteristics of the enrolled patients are presented in Table 1. The proportions of patients with young age, low BMI, advanced stages, emergency operation, positive resection margin, and receiving adjuvant chemotherapy were higher in the MAC group than in the AC group (Table 1). The chemotherapy regimens are described in Table S1.

### 3.2. Multivariate Analysis

Multivariate analysis was performed to identify differences in factors affecting the OS between AC and MAC (Table 2). The risk factors for OS in the AC group were similar to those in the MAC group. However, low and high BMI were significant risk factors for AC but not in MAC. Furthermore, the HR increased according to stage in AC but not in MAC. In the MAC group, the pathological stage III group differed significantly. However, pathologic IIA and IIB/C tumors were not observed. The effect of adjuvant chemotherapy appeared to be low for MAC. The HR of not receiving adjuvant chemotherapy in the MAC group (HR = 1.42) was lower than that in the AC group (HR = 1.63) and the *p*-value of MAC was marginal (*p* = 0.053).

### 3.3. Survival Analysis

**Figure 1:**
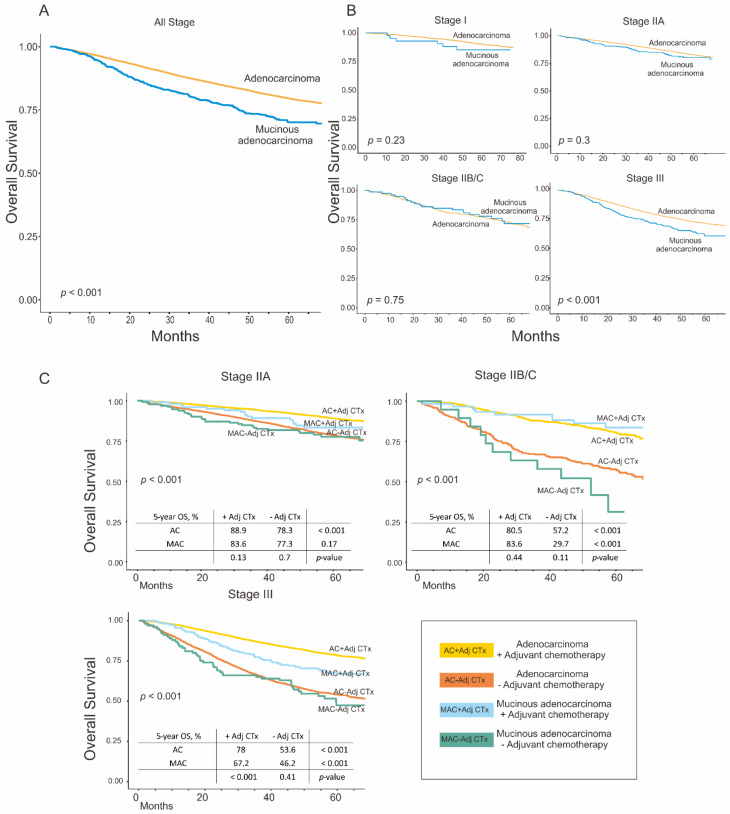
Overall survival rates in the adenocarcinoma and mucinous adenocarcinoma groups. (**A**) All stages; (**B**) according to pathologic stages; and (**C**) according to pathologic stages and the administration of adjuvant chemotherapy. Adjuvant chemotherapy showed survival benefits for patients with mucinous adenocarcinoma in stage II and III; however, the survival benefit of adjuvant chemotherapy was limited in stage III.

The OS of patients in the MAC group was worse than that of patients in the AC group (five-year OS: 79.4 vs. 70.0%, *p* < 0.001) (Figure 1A). Patients with stage I, IIA, and IIB/C disease showed no significant difference in OS between the two groups. However, stage III patients in the MAC group showed worse OS than those in the AC group (five-year OS: 71.8 vs. 61.6%, *p* < 0.001) (Figure 1B). In the subgroup analysis, patients in both the AC and MAC groups who received adjuvant chemotherapy showed better survival compared to patients who did not receive adjuvant chemotherapy, a result that was consistent in stage II and III. In stage IIA and IIB/C disease, patients with MAC administered adjuvant chemotherapy showed similar OS to that of patients with AC also administered adjuvant chemotherapy. However, in pathologic stage III, MAC patients showed worse OS than AC patients despite the concurrent administration of adjuvant chemotherapy (five-year OS: 78.0 vs. 67.2%, *p* < 0.001) (Figure 1C). Additionally, survival analysis performed on 590 patients with survival data from TCGA showed that although MAC tended to have worse survival than AC, the difference was not statistically significant (Figure S1).

### 3.4. Survival Analysis after Propensity Score Matching

**Figure 2:**
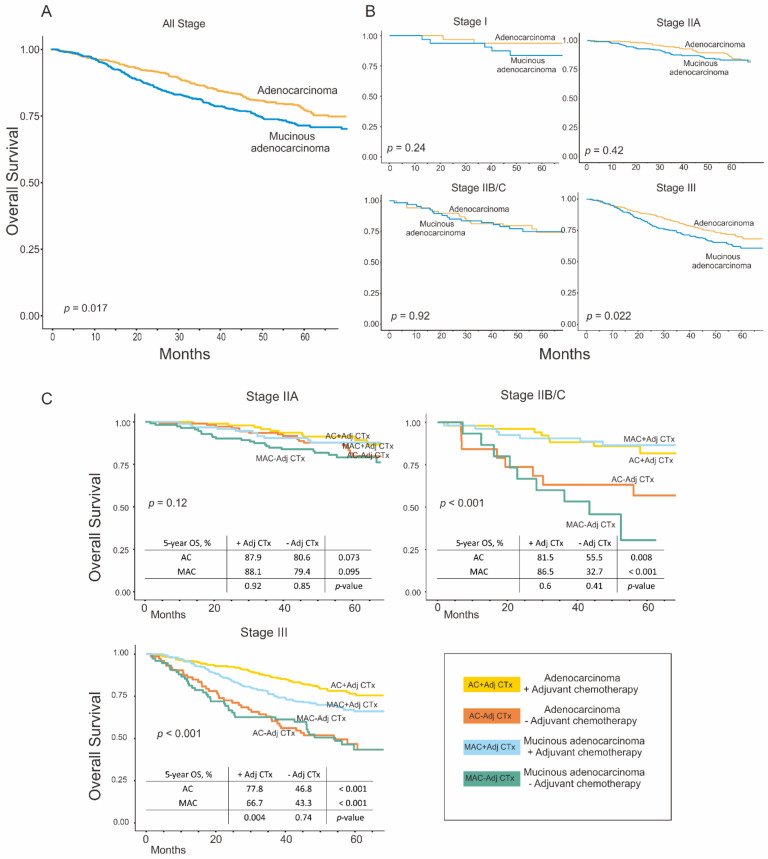
Overall survival rates in the adenocarcinoma and mucinous adenocarcinoma groups after adjustment using propensity score matching. (**A**) All stages; (**B**) according to pathologic stages; and (**C**) according to pathologic stages and the administration of adjuvant chemotherapy. The survival benefits of the adjuvant chemotherapy for patients with mucinous adenocarcinoma showed a similar trend after the adjustment except for the stage IIA patients.

Because MAC has a very low incidence compared to AC, comparing survival between the two groups may not be appropriate. In addition, as AC and MAC had significantly different clinical characteristics, the prognosis of AC and MAC were compared under the exact condition of clinical characteristics. Therefore, survival was analyzed after adjustment using propensity score matching. After adjustment, 1262 patients were selected (631 patients in each group) (Table S2) and the OS analysis was repeated (Figure 2). The results were not different from those before the adjustment, except for stage IIA (Figure 2C). Before adjustment, we observed a significant difference in the survival rates of patients with stage IIA AC chemotherapy. After adjustment, chemotherapy appeared to be effective, but the difference was not statistically significant. Additionally, we divided the matched cohort into the colon and rectal cancer groups and repeated the same analysis for each group (Figure S2). Both groups showed the same results as the initial cohort, but rectal MAC in stage III showed no survival benefits from the adjuvant chemotherapy.

### 3.5. Pathway Enrichment Analysis

**Figure 3:**
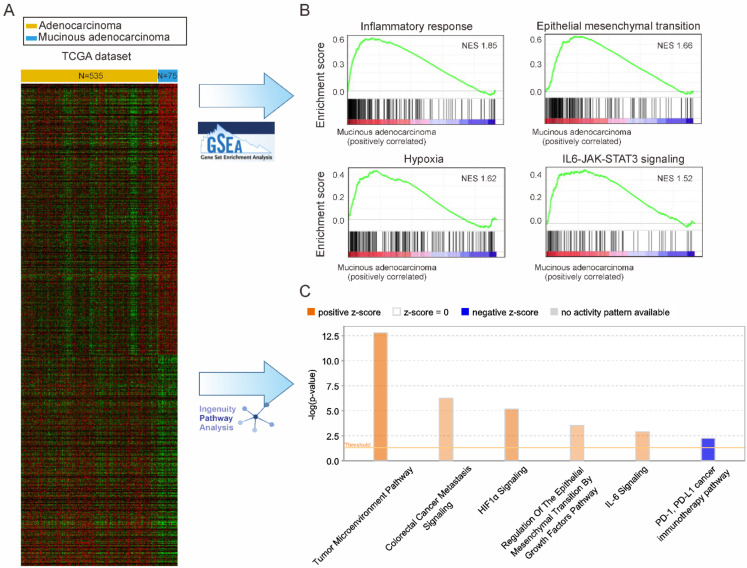
Pathway analyses of patients with mucinous adenocarcinoma. (**A**) mRNA expression from The Cancer Genome Atlas (TCGA)-provided RNA sequencing samples of 610 patients with colorectal cancer (535 adenocarcinomas, 75 mucinous adenocarcinomas); (**B**) Gene set enrichment analysis (GSEA) showing increased signaling including inflammatory response, epithelial-mesenchymal transition, hypoxia, and IL6-JAK-STAT3 signaling; (**C**) Ingenuity Pathway Analysis (IPA) showing increased signaling including tumor microenvironment signaling, colorectal cancer metastasis signaling, hypoxia-inducible factor 1-alpha (HIF1α) signaling, epithelial-mesenchymal transition, and interleukin (IL)-6 and decreased programmed death 1 (PD-1) and programmed cell death ligand 1 (PD-L1) cancer immunotherapy pathway signaling.

Survival analysis showed different effects of chemotherapy between the two groups of stage III CRC. For additional biological insight, the differences in molecular pathways according to transcriptome expression in the two groups were analyzed. RNAseq data from 610 people were obtained from TCGA and analyzed by GSEA and IPA, based on the Molecular Signatures Database (MSigDB) and Ingenuity Knowledge Base, respectively (Figure 3A). In GSEA, inflammatory response, epithelial-mesenchymal transition (EMT), hypoxia, and IL6-JAK-STAT3 signaling were increased in MAC compared to AC (Figure 3B). EMT and IL-6 signaling were also increased in IPA (Figure 3C), in addition to colon cancer metastasis signaling, hypoxia-inducible factor 1-alpha (HIF1α) signaling, and the tumor microenvironment pathway. The cancer immune therapy pathway was decreased.

### 3.6. Consensus Molecular Subtypes

**Figure 4:**
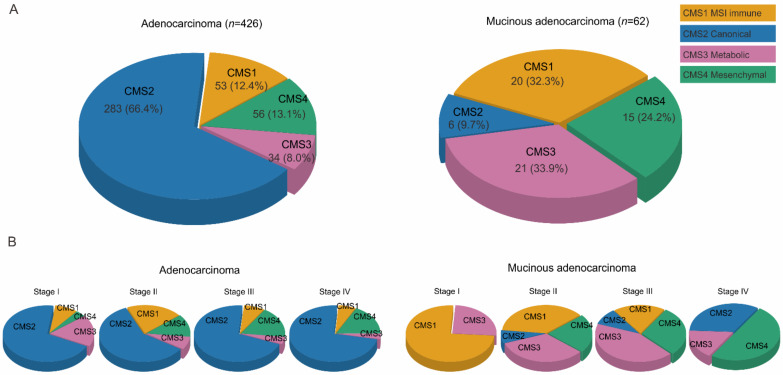
Consensus molecular subtypes (CMS) classifications of 488 samples from patients with colorectal cancer (426 adenocarcinomas, 62 mucinous adenocarcinomas). (**A**) CMS2 comprised the highest proportion in the adenocarcinoma group, compared to CMSs 1, 3, and 4 in mucinous adenocarcinoma; (**B**) CMS classification shows similar portions according to stages in the adenocarcinoma group, while the proportions of CMSs 3 and 4 increase as the stage progresses in the mucinous adenocarcinoma group.

CMS is a molecular classification system that provides meaningful biological interpretation for many colorectal cancer studies. AC accounted for most (66.4%) of CMS2. In contrast, in MAC, the proportion of CMS2 was low (9.7%) and CMS 1, 3, and 4 accounted for a significant proportion (32.3, 33.9, and 24.2%, respectively) (Figure 4). Since survival analysis showed different results according to stage, AC and MAC were classified accordingly. In AC, while CMS2 comprised the majority, no significant difference by stage was observed (Figure 4B). Surprisingly, however, there was a significant difference in the CMS ratio according to MAC stage. In stage I, the major proportion was CMS 1 (75.0%), but gradually decreased. In contrast, the proportions of CMSs 3 and 4 gradually increased in stages II and III. As the stage increased, the ratio of CMS 4 increased. Finally, in stage IV, CMS 4 accounted for 50% of samples (Supplementary Table S3).

## 4. Discussion

This study, based on a large cohort of a national registry in South Korea, showed that the OS of patients with stage III MAC was worse than that of patients with AC in the same stage. In addition, the administration of adjuvant chemotherapy was beneficial for the OS of patients with MAC at stage II and III. However, unlike for stage II patients, the survival benefit of adjuvant chemotherapy for patients with stage III MAC was limited. Pathway enrichment analysis identified clues to the biological cause of MAC chemoresistance. GSEA showed increased inflammation response, EMT, hypoxia, and IL6-Jak-Stat3 signaling in MAC, which are pathways related to chemoresistance [18,19,20,21]. IPA also showed increased EMT and IL-6 signaling, similar to the findings of GSEA, along with increase in HIF1α and colon cancer metastasis signaling, suggesting that MAC had a worse prognosis. The possible correlation between MAC and chemoresistance was supported by the CMS classification. As the stage of MAC increased, the proportions of CMSs 3 and 4, which are related to chemoresistance, increased; such findings were not observed in AC.

Many studies have compared the survival outcomes of MAC to those of AC. However, the significance of survival of the mucinous subtype of CRC remains controversial. A population-based study reported no significant difference in five-year OS between AC and MAC for the colon, rectum, and their combination [22]. Similarly, in their national cancer registry-based study, Ott et al. also reported no significant difference in survival for stage I, II, and III colon cancer [11]. Another study based on clinical data from multiple institutions also reported that even a long-term outcome of eight-year OS did not differ between MAC and AC [23]. In contrast, several studies reported that the mucinous subtype was significantly correlated with worse survival and other prognostic factors [9,10,24]. The survival outcomes of the MAC subtype were worse than those of the AC subtype, and MAC patients were younger and had more lymphatic spread, higher T stages, higher carcinoembryonic antigen levels, and right colon tumors. The MAC subtype also showed a higher inclination to peritoneal metastasis, a metastasis pattern significantly different from that of AC, which had a higher tendency for liver or lung metastasis [4,9,25,26]. One hypothesis for this tendency is that mucus under pressure allows cancer cells to spread to the peritoneal cavity easily, resulting in a higher rate of subsequent locoregional failure after surgery [27]. Another study suggested that the survival outcomes of MAC should be analyzed differently at each stage. According to a study based on the Surveillance, Epidemiology, and End Results database, stage II, III, and IV MAC showed poorer prognoses than each of the corresponding stages of AC after correcting for tumor stage [28]. In our study, MAC showed statistically worse OS than AC in all patients group. However, when compared by stage, there was no difference in OS between AC and MAC in stage I and II. Only stage III showed statistical difference.

Rectal cancer, especially mid and low rectal cancer, has different characteristics from colon cancer in the treatment including the neoadjuvant chemoradiotherapy. For this reason, previous studies have reported the prognosis of rectal cancer separately from the colon cancer group. Ott et al. showed no significant differences in OS for stages I-II AC and MAC in rectal cancer. However, stage III MAC of the rectum showed worse OS than stage III AC [11]. To evaluate the effect of rectal cancer in our cohort, we repeated the survival analysis for the colon and rectal cancer groups, respectively. Both groups showed nearly the same results as the results of the combined CRC group. We carefully suggest several reasons. The OS of rectal cancer generally followed the pathologic stages, regardless of the neoadjuvant chemoradiotherapy. Furthermore, the adjuvant chemotherapy regimen is almost the same for colorectal and rectal cancer. So, it has been assumed that colon and rectal MAC show similar patterns in terms of prognosis and the sensitivity of chemotherapy.

Some authors have reported that the mucinous subtype is not an independent prognostic factor for survival in patients with colorectal cancer [4,13,25,29] owing to the strong bias in clinical characteristics between MAC and AC patients, such as the primary tumor location, advanced stages at diagnosis, and poor grade of differentiation. These studies showed that, even when the survival of MAC patients was worse than that of AC patients, the impact on survival of the mucinous subtype was less after statistical adjustments using multivariable or propensity score-matching analysis. To verify these findings, the present study adjusted for this bias through propensity score matching. The clinical characteristics of AC and MAC differed, as previously shown; however, after matching for characteristics, the prognosis of MAC was significantly worse than that of AC overall and in stage III. Therefore, the mucinous subtype alone is an independent poor prognostic factor.

The results of the present study raise the possibility that chemoresistance is an important cause of MAC, as a factor associated with poor prognosis. In our study, subgroup analysis of its efficacy showed that adjuvant chemotherapy was beneficial in stage II and III of MAC. However, stage III MAC showed reduced responsiveness to adjuvant chemotherapy compared to other stages. When chemotherapy was administered for AC and MAC in stage III, AC showed significantly better survival than MAC. The same result was observed even after adjusting for clinical characteristics. This may be due to chemoresistance in MAC. Previous studies reported the efficacy of adjuvant chemotherapy for MAC; however, the responsiveness was reduced compared to AC [11,23,30,31]. The reduced chemosensitivity of MAC might be owing to the relatively hypoxic state due to reduced blood supply in MAC, with less microvessel density compared to AC [7]. In addition, genetic alterations associated with B-Raf (BRAF), phosphatidylinositol-4,5-bisphosphate 3-kinase catalytic subunit alpha (PIK3CA), mothers against decapentaplegic homolog 2 (SMAD2), and SMAD4, as well as microsatellite-unstable tumors, occur more frequently compared to AC, which might be associated with MAC resistance to chemotherapy [11,32].

This study analyzed transcriptome data in TCGA to determine the cause of chemoresistance in MAC. This analysis revealed increased inflammatory response, EMT, hypoxia, IL-6 signaling, and tumor microenvironment pathway expression, which were related to chemoresistance. MAC is clinically more common in inflammatory bowel diseases and Lynch syndrome [33] and the molecular secretion of mucins is associated with chronic inflammation [34]. Inflammation is closely related to hypoxia [35] and can also induce an increase in EMT and IL-6 signaling [36,37]. IL-6 is closely related to changes in the tumor microenvironment [38]. Excessive inflammation leads to T-cell depletion, making PD-1 immune chemotherapy less effective [39]. Therefore, one possible explanation for the poor prognosis and chemoresistance of MAC is that these signaling pathways are linked and constitute the molecular properties of MAC.

More surprising was that the molecular properties of MAC varied with stage. In AC, CMS2 was the most common and the overall portion did not change significantly with stage. However, in MAC, the ratio changed significantly depending on stage. The proportion of CMS1 was also higher in MAC than in AC. This may be owing to the high proportion of microsatellite instability-high (MSI-H) in MAC and the clinical characteristics that occur more frequently in the right colon. However, the proportion of CMS1 decreased as the stage increased and the proportions of CMS3 and CMS4 increased significantly. CMS3 is associated with a KRAS mutation associated with resistance to platinum chemotherapy commonly used in colorectal cancer [40]. In particular, in Stage IV, CMS4, which represents EMT, accounted for half of the cases. This explains why chemoresistance develops as the MAC stage increases and suggests that the EMT could be targeted to overcome chemoresistance in MAC. If the information on CRC stage IV was also available in the NQAP database, we could have provided the results more consistently in survival and molecular analysis. Although stage IV analysis was performed only on transcriptome analysis, this analysis would provide a clearer understanding of the chemoresistance developing as the stage increases.

This study had several limitations. First, as only insurance data were used, some important information was not available, including recurrence, T, N stage, and perineural and lymphovascular invasion. Especially, the effect on the survival of mucinous cancer with serosal exposure could not be evaluated because T4 category in stage III was not available. Second, it was not possible to subdivide the patients according to tumor-sidedness or the location of rectal cancer because the specific tumor location was not explicitly recorded. Rectal cancer may have different treatment and clinical features depending on its location. The several pieces of information associated with rectal cancer were incomplete in the NQAP database; thus, it was impossible to evaluate low rectal MAC’s unique characteristics. Third, we had to exclude missing data from the database owing to incomplete records (*n* = 20,149 (37.9% of all patients)). However, although this study excluded lot of missing data, it still analyzed an extensive dataset. Based on national registry data, the results revealed the clinical characteristics of MAC, which have been challenging to evaluate owing to its low incidence rate. Because NQAP data are created to process health insurance, they provide accurate clinical and chemotherapy information. Propensity score matching was performed for clinical and chemotherapy information in this study. Moreover, this study determined the molecular properties beyond identifying the clinical characteristics and survival of MAC.

## 5. Conclusions

The results of this study demonstrated a worse prognosis and less sensitivity to adjuvant chemotherapy for stage III MAC compared to stage III AC. This occurred owing to increased chemoresistance-related signaling, including inflammation and EMT, in MAC. EMT signaling increases as cancer progresses in MAC. Further studies on the molecular properties of MAC are needed to develop individualized treatments and overcome resistance to chemotherapy.

## Figures and Tables

**Table 1:** Patient clinicopathologic characteristics according to cell types.

	Adenocarcinoma		Mucinous Adenocarcinoma		*p*-Value
	*n* = 22,050	%	*n* = 729	%	
Age, years					0.048
<65	9096	41.3	326	44.7	
65–75	7444	33.8	215	29.5	
>75	5510	25.0	188	25.8	
Gender, male	13,073	59.3	423	58.0	0.519
BMI, kg/m^2^					0.004
<18.5	1428	6.5	64	8.8	
18.5–25	14,018	63.6	480	65.8	
>25	6604	30.0	185	25.4	
ASA classification					0.438
I–II	17,986	81.7	595	81.6	
III	3845	17.5	124	17.0	
IV	159	0.7	8	1.1	
V–VI	27	0.1	2	0.3	
Tumor location					<0.001
Colon	14,360	65.1	533	73.1	
Rectum	7690	34.9	196	26.9	
Pathologic stage					<0.001
I	4753	21.6	42	5.8	
IIA	7272	33.0	235	32.2	
IIBC	1001	4.5	79	10.8	
III	9024	40.9	373	51.2	
Number of harvested lymph nodes					0.520
≥12	18,677	95.4	598	94.8	
<12	901	4.6	33	5.2	
Positive resection margin, yes	230	1.0	17	2.3	0.002
Adjuvant chemotherapy, no	11,550	52.4	291	39.9	<0.001
Emergency operation, yes	1272	5.8	61	8.4	0.004

ASA: American Society of Anesthesiologists.

**Table 2:** Multivariate analysis of risk factors for overall patient survival according to cell types.

		Adenocarcinoma			Mucinous Adenocarcinoma		
		HR	95% CI	*p*-Value	HR	95% CI	*p*-Value
Age, years	<65	1			1		
	65–75	1.91	1.73–2.09	<0.001	1.35	0.90–2.03	0.142
	>75	3.61	3.28–4.00	<0.001	2.05	1.35–3.11	0.001
Gender	Male	1					
	Female	0.70	0.65–0.75	<0.001			
BMI	<18.5	1.45	1.31–1.61	<0.001	1.46	0.94–2.29	0.095
	18.5–25	1			1		
	>25	0.77	0.71–0.84	<0.001	0.98	0.67–1.43	0.908
ASA classification	I, II	1			1		
	III	1.56	1.45–1.68	<0.001	1.41	0.98–2.04	0.065
	IV	2.82	2.20–3.64	<0.001	2.68	0.94–7.68	0.064
	V–VI	3.26	1.68–6.32	<0.001	7.99	1.07–59.58	0.042
Location of primary cancer	Colon	1			1		
	Rectum	1.27	1.19–1.37	<0.001	1.96	1.41–2.72	<0.001
Resection margin	Negative	1			1		
	Positive	1.34	1.04–1.73	0.023	2.49	1.15–5.35	0.019
Number of harvested lymph nodes	≥12	1					
	<12	1.41	1.23–1.62	<0.001			
Pathologic stage	I	1			1		
	IIA	1.88	1.66–2.12	<0.001	1.04	0.40–2.70	0.933
	IIB/C	3.73	3.13–4.43	<0.001	2.12	0.74–6.02	0.156
	III	4.35	3.85–4.90	<0.001	3.15	1.23–8.01	0.015
Adjuvant chemotherapy, no		1.63	1.50–1.76	< 0.001	1.42	0.99–2.04	0.053
Emergency operation, yes		1.62	1.44–1.84	< 0.001	1.63	0.95–2.80	0.075

ASA: American Society of Anesthesiologists.

## Data Availability

The NQAP data presented in this study are available on request from the Health Insurance Review and Assessment Service. The data are not publicly available due to sensitive information being included. TCGA data is public and can be downloaded from the GEO and Xena browser.

## Supplementary Materials

The following supporting information can be downloaded at: https://www.mdpi.com/article/10.3390/cancers14051297/s1, Figure S1: Kaplan–Meier curves for overall survival of patients with adenocarcinoma and mucinous adenocarcinoma in The Cancer Genome Atlas (TCGA) dataset according to cancer stages (*n* = 590), Figure S2: Kaplan–Meier curves for colon and rectal cancer survival after propensity score mating, Table S1: Proportions of patients receiving chemotherapy according to chemotherapy regimen, Table S2: Clinicopathologic characteristics of patients after adjustment using propensity score matching, Table S3: Proportions of patients with consensus molecular subtypes (CMS) according to stage and histological type.
