# Supplementary material for: Prognosis and Sensitivity of Adjuvant Chemotherapy in Mucinous Colorectal Adenocarcinoma without Distant Metastasis
Source: Cancers (Basel). 2022 Mar 2;14(5):1297. doi: 10.3390/cancers14051297 (PMC8909839; doi:10.3390/cancers14051297)
Supplement: Supplementary file 1 [file cancers-14-01297-s001.zip › cancers-1559433-supplementary.pdf]

cancers

MDPI

Supplementary Material

Prognosis and Sensitivity of Adjuvant Chemotherapy in Mucinous Colorectal Adenocarcinoma without Distant Metastasis

Jun-Woo Bong [1], Jeong-An Gim [2], Yeonuk Ju [1], Chinock Cheong [1], Sun-Il Lee [1], Sang-Cheul Oh [3], Byung-Wook Min [1] and Sanghee Kang [1,*]

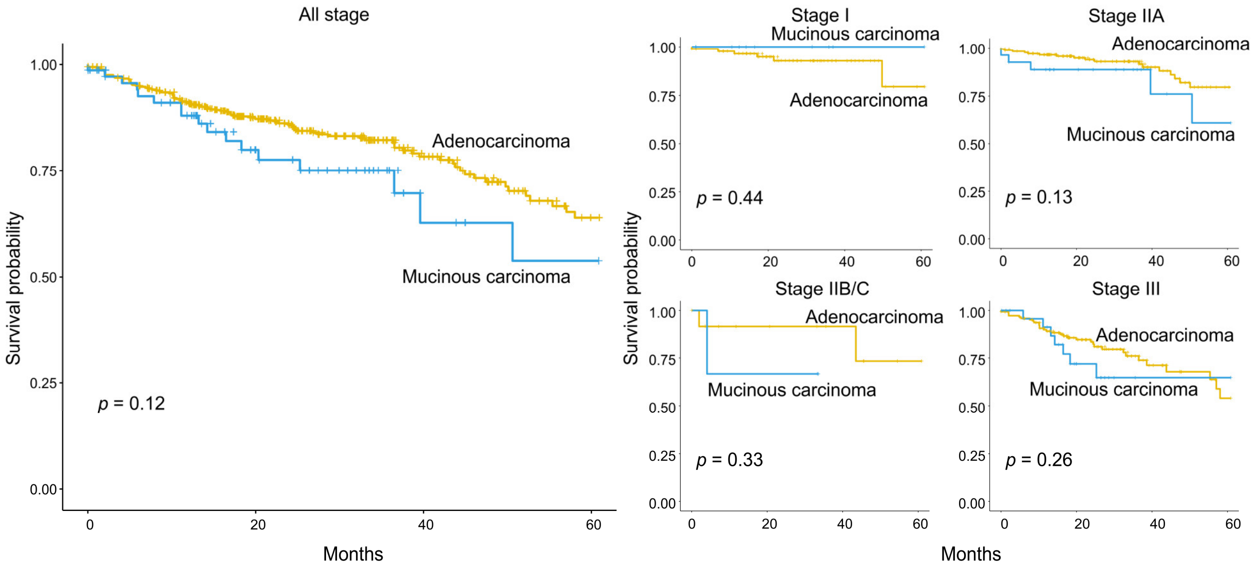

Figure S1. Kaplan–Meier curves for overall survival of patients with adenocarcinoma and mucinous adenocarcinoma in The Cancer Genome Atlas (TCGA) dataset according to cancer stages (n = 590). Mucinous adenocarcinoma showed worse survival than adenocarcinoma without statistical significance.

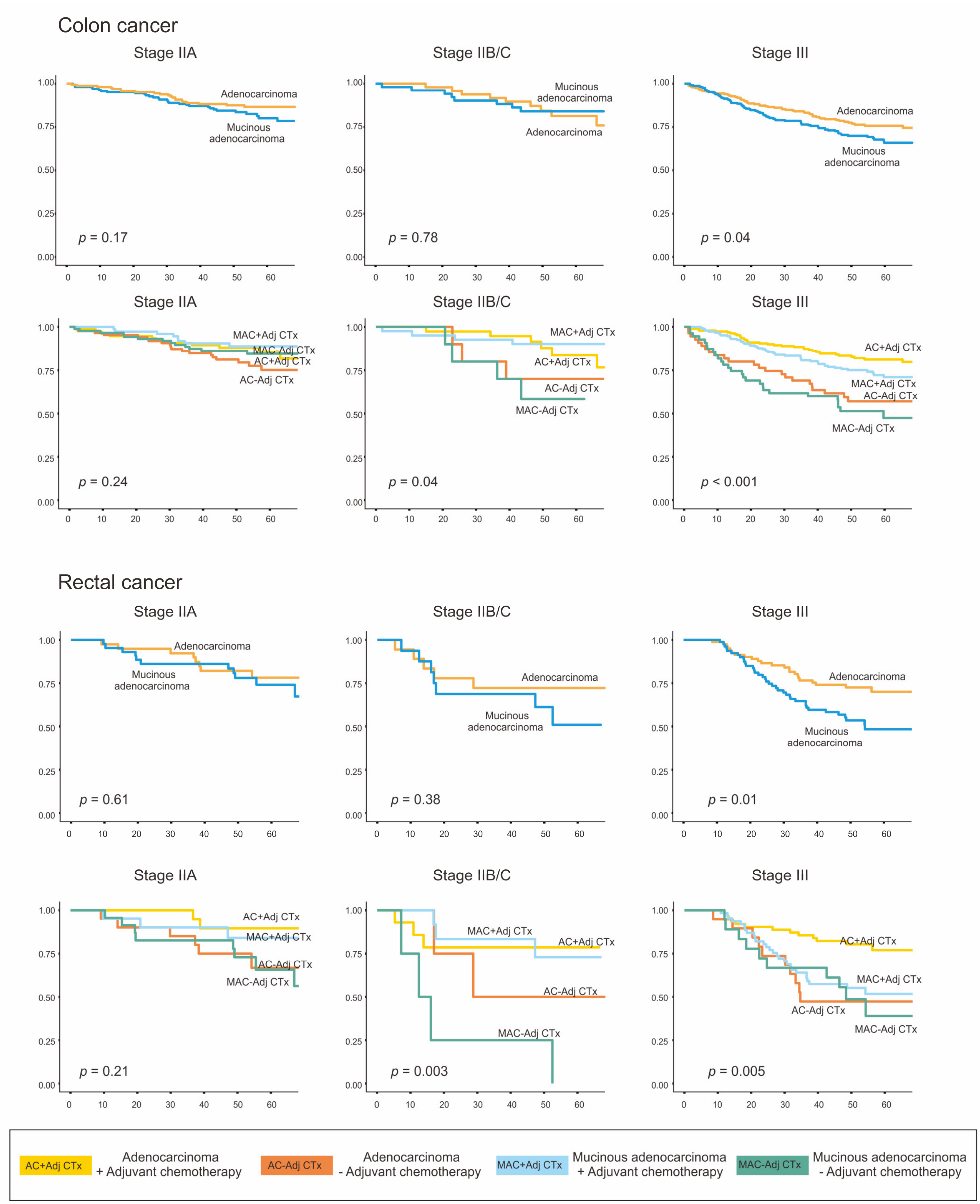

Figure S2. Kaplan–Meier curves for colon and rectal cancer survival after propensity score mating. Both groups showed similar results to the analysis results by combining colon and rectum, but rectal MAC in stage III showed no survival benefit from the adjuvant chemotherapy.

Table S1. Proportions of patients receiving chemotherapy according to chemotherapy regimen.

	**Adenocarcinoma** *n* **= 10500 (%)**	**Mucinous Adenocarcinoma** *n* **= 438 (%)**
5-FU	1959 (18.7)	62 (14.2)
Capecitabine	1414 (13.5)	47 (10.7)
Oxaliplatin	6091 (58)	275 (62.8)
Others	305 (2.9)	12 (2.7)
Unknown	731 (7)	42 (9.6)

Table S2. Clinicopathologic characteristics of patients according to cell type after adjustment using propensity score matching.

	**Adenocarcinoma**		**Mucinous Adenocarcinoma**		***p*-Value**
	n **= 631**	**%**	*n* **= 631**	**%**	
Age, years					0.980
<65	278	44.1	275	43.6	
65–75	189	30.0	192	30.4	
>75	164	26.0	164	26.0	
Gender, male	363	57.5	367	58.2	0.864
BMI, kg/m^2					0.950
<18.5	49	7.8	52	8.2	
18.5–25	422	66.9	421	66.7	
>25	160	25.4	158	25.0	
ASA classification					0.795
I–II	517	81.9	514	81.5	
III	109	17.3	111	17.6	
IV	5	0.8	5	0.8	
V–VI	0	0	1	0.2	
Location of tumor					0.893
Colon	480	76.1	477	75.6	
Rectum	151	23.9	154	24.4	
Pathologic stage					0.997
0–I	32	5.1	32	5.1	
IIA	204	32.3	207	32.8	
IIBC	70	11.1	68	10.8	
III	325	51.5	324	51.3	
Number of harvested lymph nodes					0.898

≥12	600	95.1	598	94.8	
<12	31	4.9	33	5.2	
Positive resection margin, yes	9	1.4	12	1.9	0.660
Adjuvant chemotherapy, no	232	36.8	233	36.9	1.000
Emergency operation, yes	33	5.2	35	5.5	0.901

ASA: American Society of Anesthesiologists.

Table S3. Proportions of patients with consensus molecular subtypes (CMS) according to stage and histological type.

	**Adenocarcinoma, *n* = 426**					**Mucinous Carcinoma, *n* = 62**				
		**Stage**					**Stage**			
CMS	**Total**	**I**	**II**	**III**	**IV**	**Total**	**I**	**II**	**III**	**IV**
**CMS1**	53 (12.4%)	7 (8.4%)	33 (20.4%)	9 (7.6%)	4 (6.3%)	20 (32.3%)	6 (75.0%)	10 (37.0%)	4 (19.0%)	0 (0.0%)
**CMS2**	283 (66.4%)	58 (69.9%)	96 (59.3%)	83 (70.3%)	46 (73.0%)	6 (9.7%)	0 (0.0%)	2 (7.4%)	2 (9.5%)	2 (33.3%)
**CMS3**	34 (8.0%)	15 (18.1%)	12 (7.4%)	5 (4.2%)	2 (3.2%)	21 (33.9%)	2 (25.0%)	9 (33.3%)	9 (42.9%)	1 (16.7%)
**CMS4**	56 (13.1%)	3 (3.6%)	21 (13.0%)	21 (17.8%)	11 (17.5%)	15 (24.2%)	0 (0.0%)	6 (22.2%)	6 (28.6%)	3 (50.0%)
